# P-1294. Examining the Utility of Education Alongside Low-Barrier Community Vaccination Clinics in Increasing Influenza Immunization Uptake Within Vulnerable Black Communities

**DOI:** 10.1093/ofid/ofae631.1475

**Published:** 2025-01-29

**Authors:** Jacinda Abdul-Mutakabbir, Raheem Abdul-Mutakabbir, Samuel Casey

**Affiliations:** University of California San Diego, La Jolla, California; Eastern University, San Diego, California; Congregations Organized for Prophetic Engagement, San Bernardino, California

## Abstract

**Background:**

Amidst the coronavirus disease (COVID-19) pandemic, vaccination rates for other preventable diseases, including influenza, have drastically declined across the US non-Hispanic Black population. Systemic injustice and decades of medical mistreatment have been cited as critical factors. Addressing gaps in health literacy and accessibility has been shown to promote trust and improve vaccine uptake. In this study, we aimed to utilize a multifaceted approach to investigate barriers to influenza vaccination and to promote equitable influenza immunization uptake across San Bernardino County, CA (SBC) Black communities.

**Methods:**

The intervention commenced from August to December 2023 and included a 45-minute vaccine education session followed by a community vaccination clinic. The intervention occurred one weekend per month at a pre-identified church location within SBC. Participants 18 years and older were invited to take two optional surveys before and after the education session to assess the impact of knowledge on health behaviors. Demographic data, including social vulnerability, were collected for all survey participants and those immunized in the clinic.

**Results:**

127 individuals completed the pre-survey, and 109 individuals completed the post-survey. Survey data revealed that 86% of the participants identified as Black, 75% were age 55 or older, and 98% resided in a highly vulnerable area. We identified significant positive knowledge changes when comparing baseline vaccine literacy to the knowledge gained following the presentation, particularly in areas surrounding vaccine recommendations for adults 65 years or older. Notably, 47.2% of the participants stated they were “extremely likely” to be vaccinated against influenza before receiving education. Following education, 75.4% of participants noted they were “extremely likely” to be vaccinated and 78.5% were “extremely likely” to recommend the vaccine. 96 vaccine doses (40 influenza and 56 COVID-19) were given, and 33% were co-administered.

**Conclusion:**

Our results indicate that providing education alongside vaccination can impact vaccine uptake in Black communities. Further research is needed to determine best practices for addressing limitations in vaccine uptake within minoritized populations.

**Disclosures:**

**Jacinda Abdul-Mutakabbir, PharmD,MPH**, CSL Sequiris: Advisor/Consultant|CSL Sequiris: Honoraria|GSK: Advisor/Consultant|GSK: Honoraria|Novavax: Advisor/Consultant|Novavax: Honoraria|Shionogi: Advisor/Consultant|Shionogi: Honoraria

